# Fabrication of ZnO Nanowires Arrays by Anodization and High-Vacuum Die Casting Technique, and Their Piezoelectric Properties

**DOI:** 10.3390/s16040431

**Published:** 2016-03-24

**Authors:** Chin-Guo Kuo, Ho Chang, Jian-Hao Wang

**Affiliations:** 1 Department of Industrial Education, National Taiwan Normal University, Taipei 10610, Taiwan; chinguo7@yahoo.com.tw; 2 Graduate Institute of Manufacturing Technology, National Taipei University of Technology, Taipei 10608, Taiwan; t101408074@ntut.edu.tw

**Keywords:** ZnO nanowires, anodic aluminum oxide (AAO), vacuum die casting

## Abstract

In this investigation, anodic aluminum oxide (AAO) with arrayed and regularly arranged nanopores is used as a template in the high-vacuum die casting of molten zinc metal (Zn) into the nanopores. The proposed technique yields arrayed Zn nanowires with an aspect ratio of over 600. After annealing, arrayed zinc oxide (ZnO) nanowires are obtained. Varying the anodizing time yields AAO templates with thicknesses of approximately 50 μm, 60 μm, and 70 μm that can be used in the fabrication of nanowires of three lengths with high aspect ratios. Experimental results reveal that a longer nanowire generates a greater measured piezoelectric current. The ZnO nanowires that are fabricated using an alumina template are anodized for 7 h and produce higher piezoelectric current of up to 69 pA.

## 1. Introduction

Zinc oxide (ZnO) is an n-type II-VI semiconductor group material with a wurtzite structure in a hexagonal crystal system. Since it is symmetric and has no center of symmetry, this structure has favorable piezoelectric properties [1]. As components are miniaturized, piezoelectric materials have become nanosized. In recent years, ZnO nanowires (NWs) have been used in nanogeneration devices [2,3]. In relevant works, the most representative device of this kind is the piezoelectric nanogenerator that was developed by a research team that was led by Wang [4]. First, an atomic force microscope (AFM) was used as a probe to apply stress to ZnO nanowires to make them produce strain, and then the piezoelectric current was measured. This property was further employed to develop a nanogenerator. Xue studied the response of a nanogenerator (NG) to the gas environment in which it is placed. Xue’s experimental results indicated that the output of a piezoelectric nanogenerator (NG) that was fabricated using ZnO nanowire arrays is largely influenced by the density of the surface charge carriers at the nanowire surfaces [5]. Lin developed Pd nanoparticles that were uniformly loaded on the whole surfaces of ZnO nanowire arrays using a simple hydrothermal method. The piezoelectric output that was generated by the Pd/ZnO nanoarray nanogenerator acted not only as a power source but also a response signal to ethanol at room temperature [6]. Fu fabricated a ZnSnO_3_/ZnO nanowire piezo-nanogenerator as a self-powered gas sensor with high sensitivity, selectivity, and reliability to detect liquefied petroleum gas at room temperature. The results indicated that the sensitivity of the self-powered gas sensor to 8000 ppm liquefied petroleum gas was up to 83.23, and the limit of detection was 600 ppm [7]. Zeng utilized a low-temperature hydrothermal method to fabricate vertically aligned ZnO nanowires that were grown on graphene oxide (GO) films. The results indicated that the graphene oxide layer facilitated the vertical growth of ZnO NWs and improved their crystal quality [8]. Lin used a solution-free, catalyst-free, vapor-phase growth method to synthesize ZnO nanorod arrays on Al-doped ZnO (AZO) films. The experimental results of Lin revealed that the electrical performance of the transparent conductive oxide films was not degraded by the growth of nanorod arrays, and the nanorods were highly crystalline [9]. Tian performed pure Zn chemical vapor deposition on an anodic aluminum oxide substrate at 950 °C to form develop multilayer comb-like zinc oxide nanostructure. The experimental results of Tian reveal that the growth of the ZnO nanostructures is controlled by the vapor-solid (VS) growth mechanism and is a diffusion-limited process [10].

To fabricate a one-dimensional nanostructure, Zhang *et al.* successfully placed bismuth (Bi) in a vacuum chamber for heating and melting, and cast the molten Bi into the alumina template using high-pressure gas [11,12]. A high temperature was used to melt the metal. The molten metal material was cast into the nanotemplate by pressurization to fabricate arrayed nanowires. In the fabrication of the nanowires, the control variables were pressure value, heating temperature, the properties of the liquid metal, and the applied force. However, the pressure of the high-pressure gas was limited by a gas compressor. To solve the problems that were caused by gas injection, mechanically powered hydraulic equipment was used to apply additional pressure in a newly developed high-vacuum die casting technique that combined traditional casting with a nano-technique [13].

Anodization was used to fabricate porous anodic aluminum oxide (AAO) templates. The pore size of the template was controlled using various solutions. Accordingly, highly regularly arrayed nanopores were formed [14]. The depth of the pores was determined by the anodizing duration. Then high-vacuum die casting technique was used to cast the material into the nanopores of the template, and thus to fabricate nanowires of different lengths. The purpose of this work is to fabricate ZnO nanowire with a diameter of 80 nm inside the nanopores of an AAO template and to study the relationship between the length of the ZnO nanowire and the piezoelectric properties of ZnO material with a one-dimensional nanostructure. The results of this research may improve our understanding of ZnO material on the nanoscale.

## 2. Experimental Details

**Figure 1:**
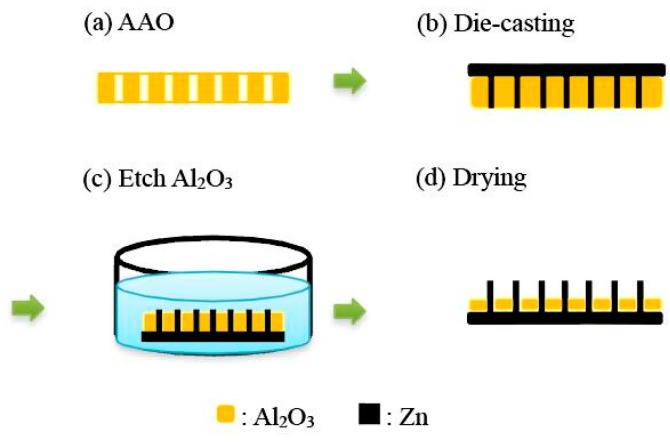
Experimental process. AAO: anodic aluminum oxide.

In the experiment herein, porous AAO film was used as a template, on which zinc foil was placed. The template was heated until molten. Normal pressure was applied to cast the molten zinc into the nanopores of the template. Atmospheric annealing was performed to transform zinc nanowires into ZnO nanowires. Finally, part of the AAO nanotemplate was removed to expose the nanowires. Figure 1 shows a flow chart of the experiment.

**Figure 2:**
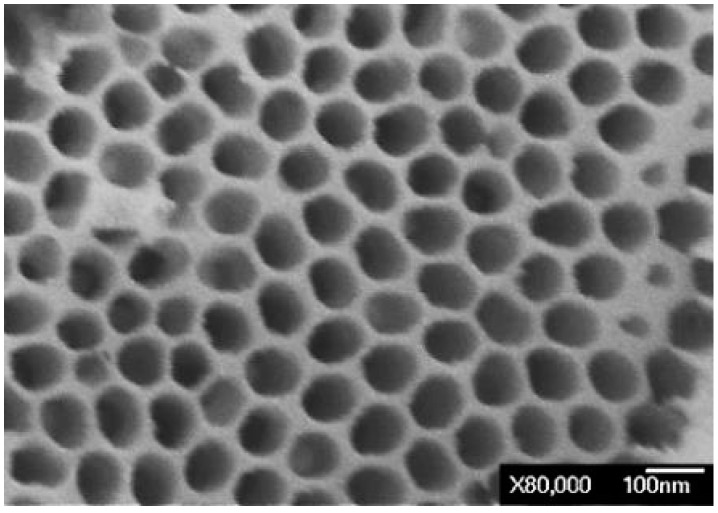
SEM image of alumina template with pores of size 80 nm.

In 1995, Masuda *et al.* discovered the self-assembling porous alumina film, and developed hexagonally arrayed porous alumina [15]. They used a two-step anodization method to grow alumina film. Aluminum foil with a purity of 99.7% was used as the substrate. HClO_4_, C_2_H_5_OH, and HOCH_2_CH_2_OC_4_H_9_ were mixed uniformly in a ratio of 3:14:3 to form an electrolytic polishing solution. With pure aluminum foil as the anode and graphite foil as the cathode, electrolytic polishing of the foil was performed, completely flattening, lustering, and smoothing its surface, favoring the fabrication of the AAO film with very regularly arrayed nanopores. Thereafter, 0.3 M oxalic acid solution was used in two-phase anodization. With aluminum foil as the anode and graphite foil as the cathode, 40 V voltage was applied for 1 h to perform phase-1 anodization. Then, 6% phosphoric acid solution was added to 2% chromic acid solution, and the resulting solution was used to remove the AAO film that was grown in phase-1 at 60 °C. Finally, phase-2 anodization was performed. A second anodization for 5, 6, and 7 h yielded films of various thicknesses. As presented in Figure 2, the nanopores in the obtained AAO template were very regularly arrayed with a complete structure, and all pores had a size of 80 nm.

**Figure 3:**
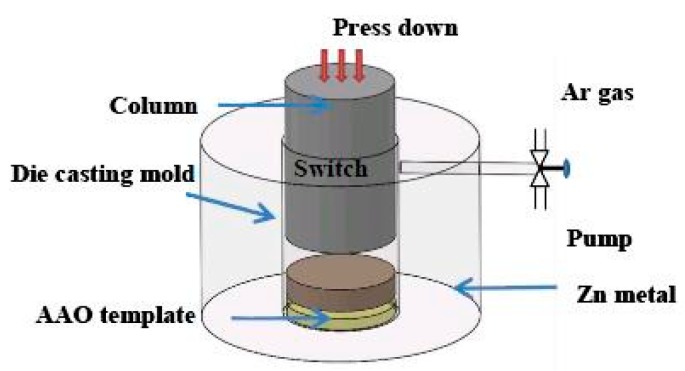
Die casting mold.

The nanopores in the AAO fabricated in the experiment are highly regularly arrayed. The diameter of the pores is 80 nm, and the thickness of the tube wall increases with time. The template helps in fabricating nanowires, supporting good regularity. The AAO template and zinc film were placed in the die cast mold, as presented in Figure 3. The mold was depressurized to a vacuum of 10^–3^ torr, and heated at 750 °C for 60 min. Then, a hydraulic force (kgf/cm^2^) was applied to cast the molten zinc into the nanopores of the AAO template. A period of condensation yields zinc nanowires.

Let the applied pressure of the liquid metal that enters the AAO template be proportional to the surface tension of the liquid metal. The surface tension of zinc in the liquid state at 600 °C is 787 dyne/cm [16]. A hydraulic force was applied to overcome the surface tension of the liquid; the pressure of the liquid that enters a nanotube was calculated using Equation (1), where F denotes the normal force; A is the surface area of the sample; γ is the surface tension of the liquid; θ is the contact angle between the liquid and the solid, and r is the diameter of the microtubules. As measured by a contact angle analyzer, the θ of AAO was 104.85°. The diameter of the AAO tube was 80 nm. The surface area of the sample was 3.14 cm^2^. Therefore, the additionally applied force of zinc liquid entering the critical side of AAO was 3.16 × 10^8^ dyne.


P = F/A = –2γ (cosθ/r)
(1)

The zinc nanowires, following die casting, were placed in an atmospheric heat treatment furnace. The temperature of the furnace was increased to 300 °C and maintained for 36 h to carry out oxidization to completely transform the zinc into zinc oxide nanowires [17]. A part of the AAO template was removed using 0.1 M sodium hydroxide (NaOH) solution to expose the nanowires. Since part of the template remains, the nanowires maintain their verticality, and the overall structure of the nanowires array has enough strength to facilitate the subsequent measurement of its piezoelectric properties. Finally, deionized water was used to perform ultrasonic cleaning to remove the residues after the reaction of the AAO template with NaOH. Bake drying was performed on a heating platform. This concludes the fabrication of the ZnO nanowire piezoelectric component.

This paper uses SEM to observe the thickness of the AAO template, and an X-ray diffractometer (XRD) to analyze the crystal structure of nanowires. This study verifies that if nanowires are completely transformed into ZnO nanowires, then the crystal direction of ZnO nanowires of three lengths can be observed. Finally, a conductive atomic force microscope (C-AFM) is used to scan the nanowires in contact mode and measure the piezoelectric current. The length, width, and height of the AFM cantilever are 160 µm, 50 µm, and 4.6 µm, respectively. The height and radius of the probe are 14 µm and 7 nm, respectively.

## 3. Results and Discussion

**Figure 4:**
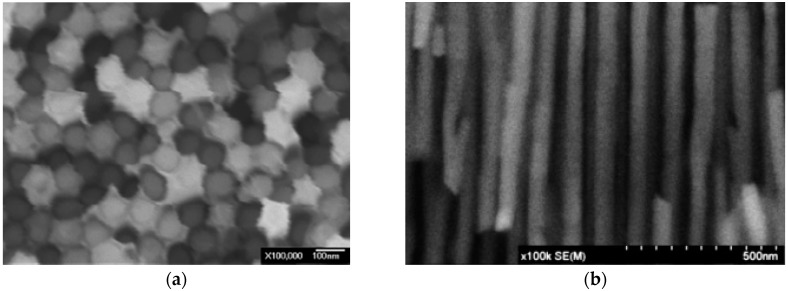
SEM images of nanowires that are cast into an AAO template: (**a**) top view; (**b**) lateral view.

The surface morphology of nanowires is observed by scanning electron microscopy (SEM). Figure 4 shows the SEM images of a nanowire that is cast into an AAO template.

**Figure 5:**
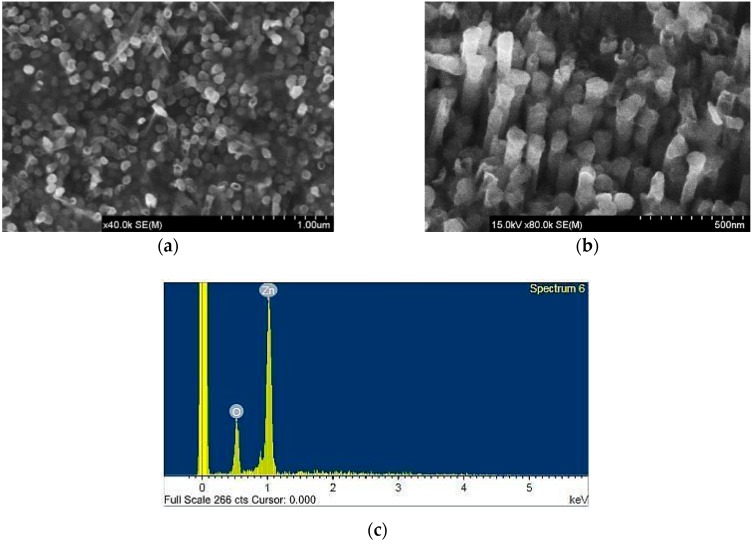
SEM images of ZnO nanowires: (**a**) top view; (**b**) lateral view, and (**c**) EDS pattern.

**Figure 6:**
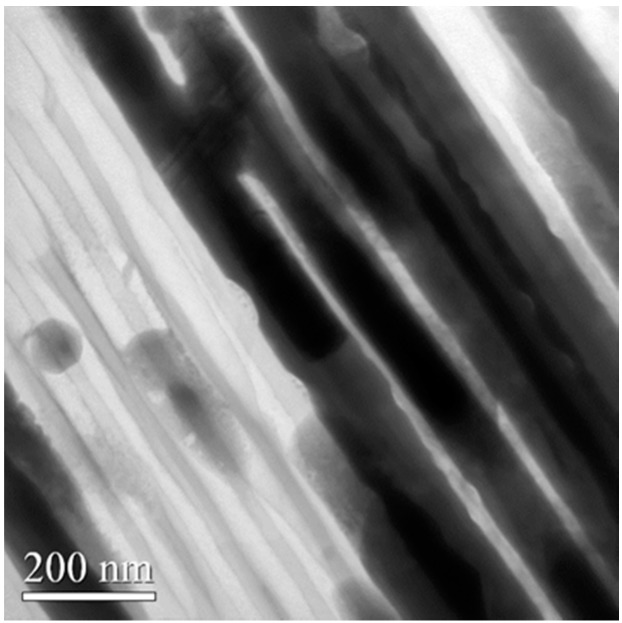
TEM image of prepared ZnO nanowires.

Figure 5a shows the SEM front image of the partly removed AAO template. Figure 5b displays the SEM image of the sample that is slanted 30°. In the figures, the well distribution of ZnO nanowires array can be observed. The ZnO nanowires are found to be very densely arrayed, and each exhibits good verticality. The SEM images in Figure 5a,b reveal that the diameter of the prepared ZnO nanowires is 80 nm and these nanowires are primarily well aligned on the bottom of the AOO template. Figure 5c presents the energy dispersive spectrometer (EDS) analytic chart. The area of EDS analysis is indicated by the blue square in Figure 5a. Table 1 presents the constituent elements of the fabricated nanowires. The table reveals that atmospheric annealing can transform zinc into ZnO nanowires. Additionally, to elucidate the crystalline quality of the prepared ZnO nanowires, Figure 6 presents a TEM image thereof. The prepared ZnO nanowires are single-crystal ZnO with a wurtzite structure and a <0001> growth direction.

**Figure 7:**
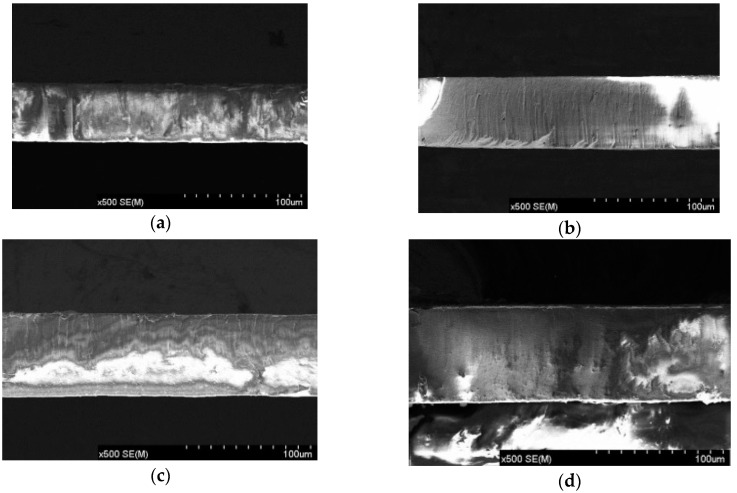
SEM images of the cross-section of alumina templates that were anodized for 5–8 h: (**a**) 5 h, (**b**) 6 h, (**c**) 7 h, and (**d**) 8 h.

**Figure 8:**
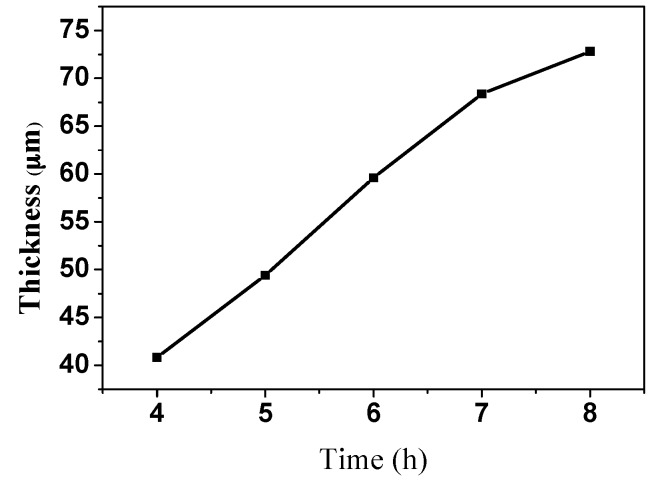
Relationship between thickness of alumina template and anodizing duration.

As shown in Figure 7, the AAO templates that were anodized for 5–8 h had thicknesses of 49.4 μm, 59.5 μm, 68.1 μm, and 72.6 μm. The thickness of each template was determined by the duration of the second anodization. Figure 8 plots the relationship between the thickness of the template and the anodizing duration. When the anodizing time was 5–7 h, the thickness increased at a rate of 9–10 μm/h, and when the anodizing time was 7–8 h, the thickness increased at a rate of 4–5 μm/h, because when the thickness of the template reached a certain value, the electrolyte could easily reach the bottom of the pores, so the growth rate was reduced.

**Figure 9:**
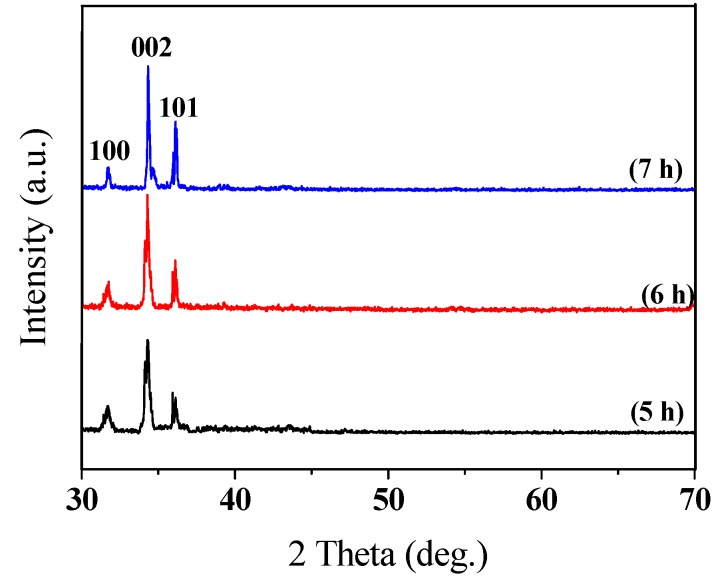
XRD patterns of ZnO nanowires that were fabricated using alumina template that was anodized for 5–7 h.

Figure 9 presents XRD diagrams that were obtained at 0.02° intervals of diffraction angle 2θ from 30° to 70°, following the XRD measurement of the ZnO nanowires that were made using the AAO template that was anodized for 5–7 h. When the diffraction angle 2θ was approximately 34.3°, the ZnO nanowires of all three lengths yielded a very strong diffraction peak. Analysis of of the XRD chart and a comparison with JCPDS database revealed that the peak value was generated by the ZnO of the (002) side. Therefore, the ZnO nanowires that were fabricated in this work exhibit preferentially c-axis-oriented growth, and the peak strength tends to be proportional to the length of the nanowire. The chart also reveals that Zn nanowires have undergone heat treatment at a fixed temperature of 300 °C for 36 h are completely oxidized and thus transformed into ZnO nanowires.

ZnO exhibits non-ferroelectric piezoelectricity. Therefore, it cannot generate piezoelectricity by polarization, and only a structure that grows along the c-axis is piezoelectric. Therefore, preferentially c-axis-oriented ZnO is just highly suitable for fabricating piezoelectric components.

**Figure 10:**
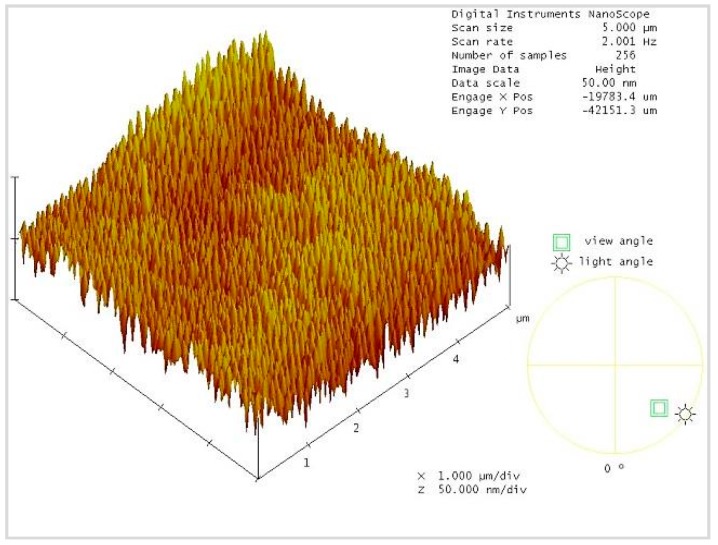
3D diagram of surface morphology of ZnO nanowires, obtained using atomic force microscopy (AFM).

AFM in contact mode was used to measure the surface morphology over 5 μm × 5 μm areas of the surfaces of nanowires that were fabricated by the above experimental method. As shown in Figure 10, the surface morphology of the ZnO nanowires is very densely arrayed, and each ZnO nanowire exhibits good verticality.

**Figure 11:**
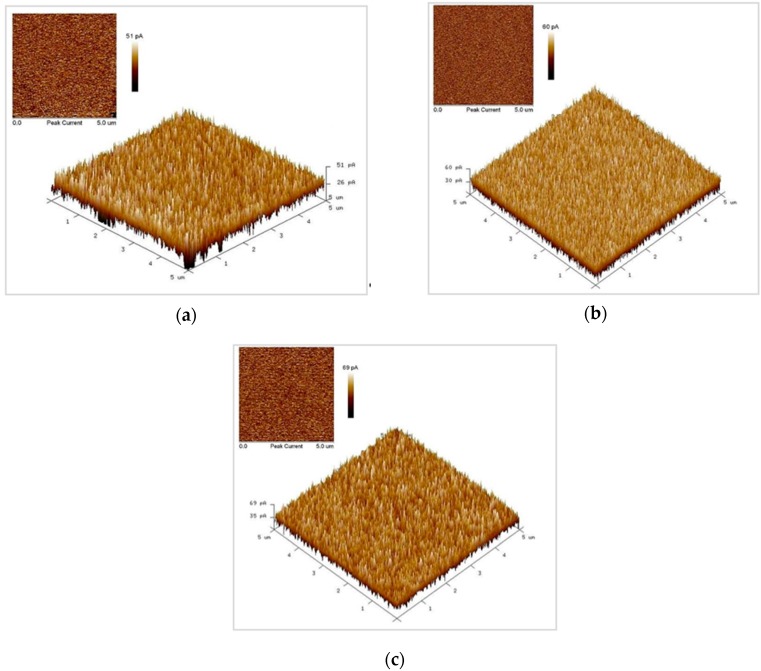
Piezoelectric current diagrams of ZnO nanowires, fabricated using alumina template that was anodized for 5–7 h, measured using conductive atomic force microscopy (C-AFM): (**a**) 5 h, (**b**) 6 h, and (**c**) 7 h.

Conductive AFM was used to measure the piezoelectric currents of the ZnO nanowires of three lengths. Through the probe, stress was applied to deform the nanowires. The piezoelectric current that was generated on a certain area of each nanowire was measured. When a current was generated on a surface, the probe received a signal. An image of the current distribution on the scanned area was obtained. The current/voltage curve (*I*–*V* curve) at a particular point was obtained. Figure 11a–c plot the piezoelectric currents on ZnO nanowires that were fabricated using the alumina template that had been anodized for 5–7 h, obtained using C-AFM. As shown in the figures, the piezoelectric current increased with the length of the nanowire. The nanowire with a length of approximately 70 μm that was fabricated after anodization for 7 h generates a current of 69 pA. Nanowires cause a deflection of a probe that is applied to stress them under scanning process. As the length of the nanowires increases, the probability of a deflection increases, and larger piezoelectric currents are measured.

**Figure 12:**
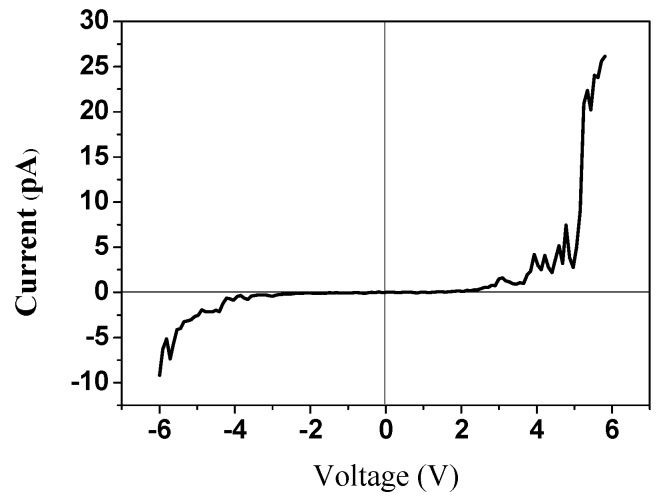
Current/voltage properties of ZnO nanowires, measured using a platinum-plated probe that serves as a metal electrode.

Figure 12 plots the current/voltage property curve of ZnO nanowires measured using a platinum-plated probe serving as a metal electrode. Since Schottky contact exists between ZnO nanowires and metal electrodes, the current/voltage curve of ZnO nanowires has an asymmetric property and exhibits the property of a diode. Therefore, a piezoelectric component that is fabricated from ZnO generates a direct current.

## 4. Conclusions

In this work, AAO templates were made, the formation of nanopores was controlled, and a high-vacuum die casting technique was used to cast zinc into the nanopores of AAO. Zinc was transformed into ZnO nanowires by atmospheric heat treatment, and the AAO template was then removed to expose the nanowires. Microstructural analysis was carried out and observations made. Finally, the piezoelectric current that was generated by ZnO nanowires was measured using C-AFM. The results of this work are summarized as follows:

1. An aluminum template with a purity of 99.7% was anodized to produce an AAO template. The nanopores in the template were highly regularly arrayed and had a high aspect ratio. Finally, process parameters were optimized to minimize the cost of the required consumable materials.

2. The stress associated with the capillary phenomenon in die casting was calculated to obtain the normal force that was required to cast molten zinc metal into nanopores. The pressure was adjusted by using the controller of the die casting machine. A hydraulic force was used to cast molten zinc into the AAO template. After atmospheric heat treatment, arrayed ZnO nanowires were obtained.

3. The nanowires that were fabricated using the AAO template were very dense, had an aspect ratio of over 600, were well arrayed, and exhibited good verticality.

4. The arrayed ZnO nanowires that were fabricated herein exhibited preferentially c-axis-oriented growth. The (002) peak strength was proportional to the length of the nanowire.

5. As observed from the test of piezoelectric properties, elucidated using C-AFM, longer nanowires generated a greater measured piezoelectric current. Among these ZnO nanowires, those fabricated using the alumina template that had been anodized for 7 h generated the greatest piezoelectric current of 69 pA.

## Figures and Tables

**Table 1:** EDS analytic composition of ZnO nanowire.

Element	Weight %	Atomic %
O K	14.44	40.82
Zn L	85.56	59.18
Total	100.00	100.00
